# Vici syndrome with pathogenic homozygous EPG5 gene mutation: A case report and literature review

**DOI:** 10.1097/MD.0000000000022302

**Published:** 2020-10-23

**Authors:** Kamal T. Abidi, Naglaa M. Kamal, Ayman A. Bakkar, Saad Almarri, Rehab Abdullah, Maram Alsufyani, Arwa Alharbi

**Affiliations:** a Associate Professor of Pediatrics and Pediatric Nephrology. Faculty of Medecine, Al Manar University, Tunis, Tunisia; b Professor of Pediatrics and Pediatric Hepatology, Pediatric Department, Kasr Alainy Faculty of Medicine, Cairo University, Cairo, Egypt; c Consultant Pediatric Endocrinologist; d Pediatric Resident; e Family Medicine Resident, Alhada Armed Forces Hospital; f Medical Student, Faculty of Medicine, Taif University, Taif, KSA.

**Keywords:** epg5, vici syndrome

## Abstract

**Rationale:**

Vici syndrome (VICIS) is a rare, autosomal recessive neurodevelopmental disorder with multisystem involvement characterized by agenesis of the corpus callosum, congenital cataracts, cardiomyopathy, combined immunodeficiency, significant developmental delay, and hypopigmentation and in some cases loss of hearing. It is caused by mutations in Ectopic P-granules protein 5 gene, which is responsible for regulating autophagy activity.

**Patient concern:**

We report a 6-month-old Saudi female patient who was the second-born baby of first cousins. She was born by normal spontaneous vertex vaginal delivery. Parents noticed that she had global developmental delay and recurrent hospital admissions due to chest infections.

**Diagnosis:**

Brain magnetic resonance imaging showed brain atrophy with corpus callosum agenesis. Ophthalmology examination revealed bilateral congenital cataract. Molecular genetic testing identified the pathogenic homozygous variant c.4751T>A p. (Leu1584∗) on exon 27 of the EPG5 gene and confirmed the diagnosis of Vici syndrome.

**Interventions:**

Supportive multidisciplinary care plan was initiated to this untreatable syndrome.

**Outcomes:**

The patient died at the age of 6 months due to sepsis with uncompensated septic shock.

**Lessons:**

VICIS is a rare untreatable disorder with worldwide distribution. High index of suspicion is needed to diagnose it and family genetic counselling is crucial.

## 1 Introduction

VICIS is one of the rare autosomal recessive disorders, belonging to the group of congenital disorders of autophagy. Individuals born with this disorder inherit 2 copies of the defective gene on the same autosome, one from each parent. The prevalence of VICIS is less than 1/1,000,000 worldwide. Disease onset can start since antenatal or neonatal age and the age at death can range from stillbirth to infantile, early or late childhood.^[1]^ Byrne et al^[3]^ identified 30 families with members that have VICIS. Of these, 2 families were from Saudi Arabia, 2 were from United Arab Emirates, 2 were Israeli-Arabs, one was Egyptian, and one was Omani. The exact pathophysiological mechanism underlying VICIS is yet to be understood.

The syndrome presents with a wide range of presentations involving multiple systems^[2,3]^ and is caused by a mutation in ectopic P-granules protein 5 gene (EPG5) in chromosome 18.^[3]^ The EPG5 protein is responsible for regulating autophagy activity, a pivotal mechanism for the development and proper functioning of body organs. The first 2 cases were described by Vici and his colleagues in 1988. Vici was an Italian physician who reported 2 siblings with a set of clinical features comprising agenesis of the corpus callosum, cutaneous hypopigmentation, bilateral cataract, cleft lip and palate, and combined immunodeficiency.^[4]^

Since that original description of the disorder, an increasing number of cases have been reported with almost 78 confirmed cases published to the date.^[4–23]^

The patients have presented, mostly in infancy, with characteristic features of VICIS together with other phenotypic features such as progressive failure to thrive, microcephaly, nystagmus, dysmorphic features, cardiomyopathy, hypotonia, recurrent pulmonary infections, and variable immunodeficiency, among others.^[5]^ In this report, we describe a 6-month-old Saudi female infant diagnosed with VICIS features and global developmental delay who was proved genetically.

## 2 Case description

JA was a 6-month-old female patient who was the second baby of a consanguineous parents. Her elder sibling is apparently healthy 5-years-old girl. JA was born by normal spontaneous vaginal delivery after full term pregnancy and her birth weight was 3 kg. Her mother had gestational diabetes mellitus and she was on diabetic diet plan. No history of neonatal intensive care unit admission.

Although parents were well educated with some physicians being family members and despite, they noticed that their baby is not developing normal and is being hypotonic, but they denied this fact until the age of 4 months.

At the age of 4 months, their baby started to have respiratory distress with desaturation and was admitted as a case of acute bronchiolitis. Her assessment revealed; global developmental delay, hypotonia with head lag, microcephaly and micrognathia. There was no associated hypopigmented skin or hair. Her abdominal examination was normal with normal female external genitalia.

During hospital stay, the patient was noticed to have weak sucking and swallowing with recurrent chocking. Gastroesophageal reflux disease was investigated with no abnormalities. On the 8th day of hospitalization, the patient developed frequent tonic-clonic seizures with up rolling of both the eyes. Her electroencephalography revealed an abnormal focal epileptic discharge and levetiracetam was started.

**Figure 1:**
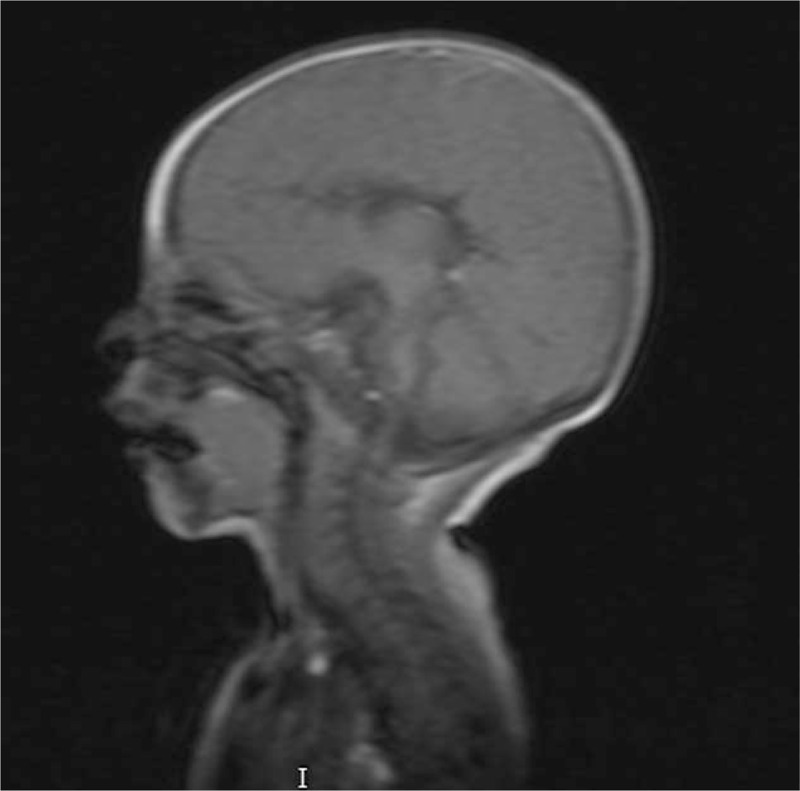
Brain Magnetic resonance imaging showing corpus callosum agenesis.

Her routine laboratory investigations were normal, and her immunological work up revealed normal immunoglobulin level. Ophthalmological examination revealed bilateral congenital cataract. Brain magnetic resonance imaging showed brain atrophy with corpus callosum agenesis (Fig. 1) and poor grey and white matter differentiation with a scarce myelin sheath. Her echocardiography showed hypertrophic cardiomyopathy with mitral regurgitation, tricuspid regurgitation, and pulmonary hypertension (Fig. 2 ). Chromosomal studies revealed a normal karyotype (46XX).

**Figure 2:**
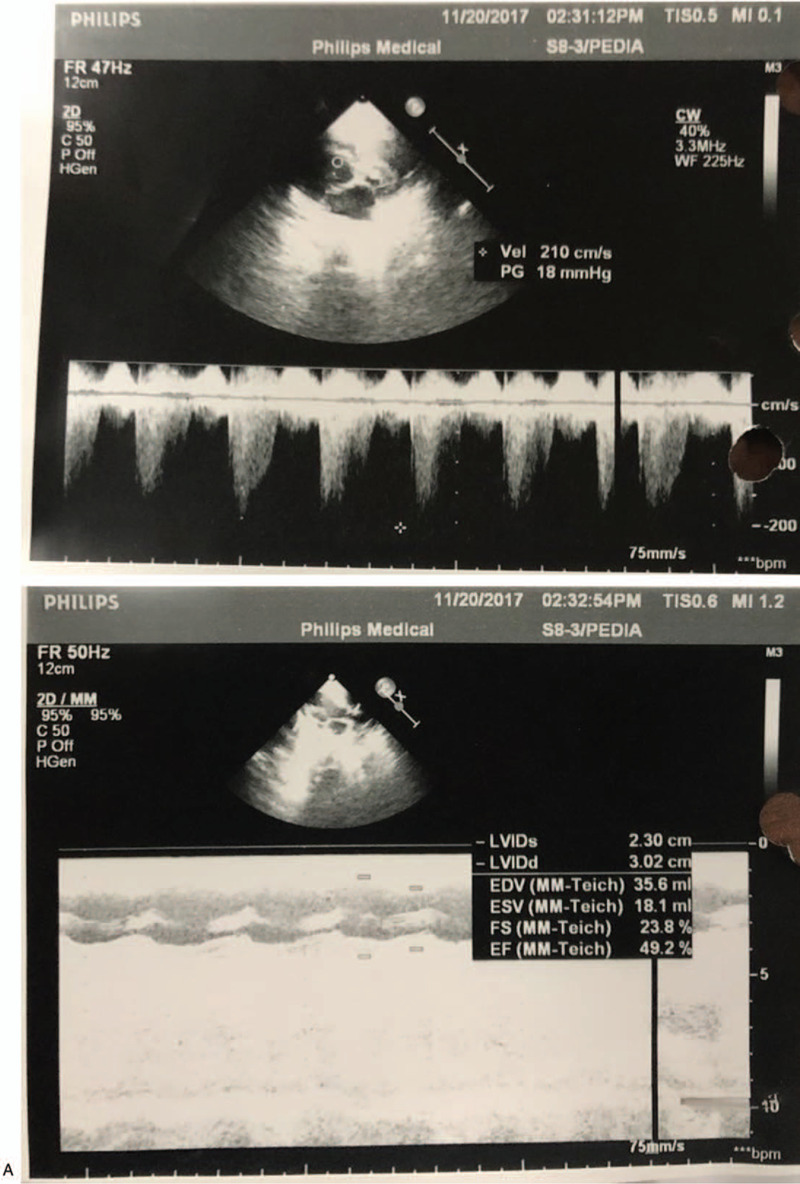
Echocardiography of the patient showing hypertrophic cardiomyopathy with mitral regurgitation, tricuspid regurgitation, and pulmonary hypertension.

**Figure 2 (Continued):**
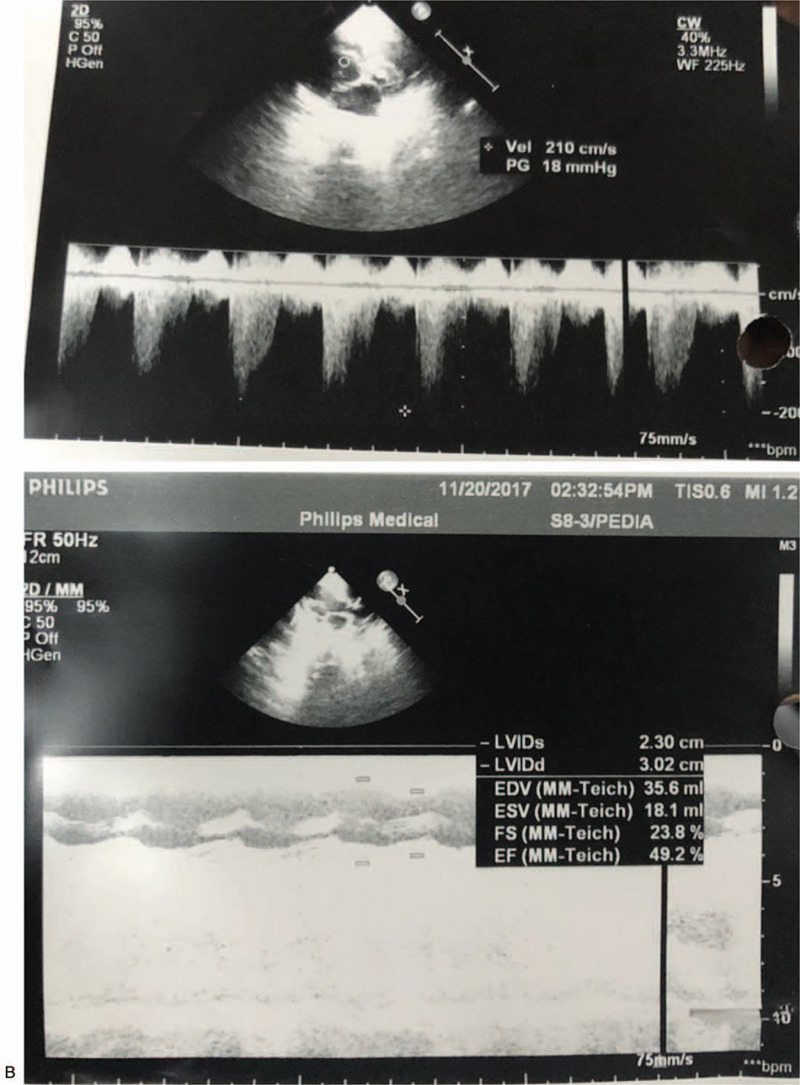
Echocardiography of the patient showing hypertrophic cardiomyopathy with mitral regurgitation, tricuspid regurgitation, and pulmonary hypertension.

Based on the constellation of findings in the history, physical examination and investigations; the diagnosis of VICIS was suspected.

**Figure 3:**
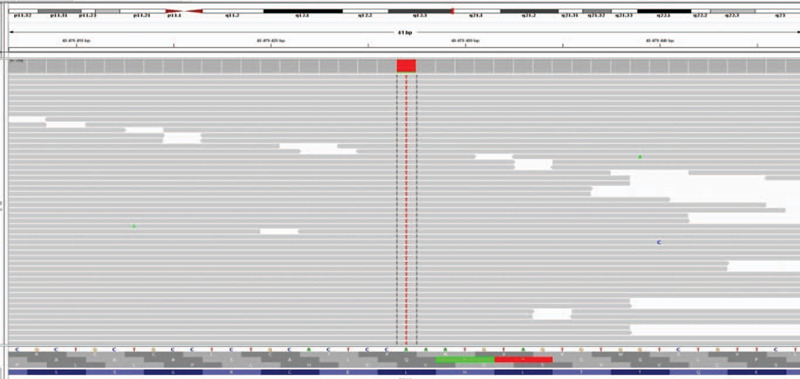
Whole exome sequencing of the patient.

Molecular genetic testing with whole exon sequencing identified the pathogenic homozygous variant c.4751T>A p. (Leu1584∗) on exon 27 of the EPG5 gene and confirmed the diagnosis (Fig. 3).

Genetic counseling for the patients parents was carried out and the family was tested to the detected mutation which identified that both parents are heterozygous and that her sister was normal. Parents were advised for pre-conception counseling and to consider in vitro fertilization treatment.

The patients terminal event was at the age of 9 months when she presented to the emergency room in critical condition and was shifted to the pediatric intensive care unit as a case of refractory septic shock with cardiac failure. Resuscitative measures were done but the patient did not revive and declared dead.

## 3 Discussion and conclusions

The infant reported here was the 3rd case reported VICIS from Saudi Arabia. Since the discovery of VICIS in 1988, a total of 79 cases including our patient have been reported. We summarized all the common features that have been associated with all patients reported with VICIS to the date in Table 1.

**Table 1:**
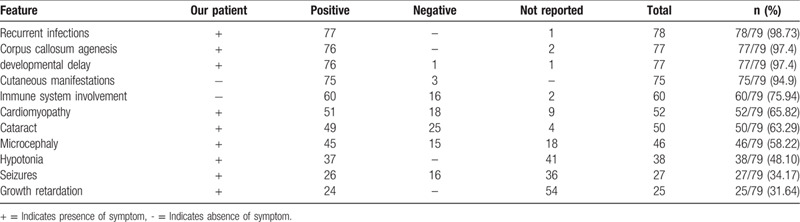
Common clinical features of 78 cases of Vici syndrome.

Most of the reported cases were associated with recurrent infections (98.73%), agenesis of corpus callosum (97.4%), developmental delay (97.4%), skin involvement (94.9%), immunodeficiency (75.94%), cataract (63.2%), and cardiomyopathy (65.8%) with the hypertrophic type being the most commonly reported.

Our findings support those published in a review of 38 cases^[3]^ of VICIS where agenesis of corpus callosum, profound developmental delay, and immune problems were the most common shared features.

Congenital cataract either unilateral or bilateral is considered as one of the classical diagnostic features of VICI syndrome. Other ocular manifestations such as optic neuropathy, nystagmus, and mild ptosis have been reported. In our case, the patient had a bilateral congenital cataract. Besides clinical assessment, there are many investigations to help shorten the list of differential diagnoses and assess the extent of organ involvement.^[24,25]^ For imaging, a brain Magnetic resonance imaging is essential to detect agenesis of corpus callosum and other less specific neuroradiological abnormalities that have been reported, such as vermis and pons hypoplasia.^[1–4]^ Chest X-ray and echocardiography are useful to detect lung and cardiac involvement, respectively. Abdominal ultrasonography helps to confirm laboratory findings, whether abdominal organs are affected or not.

To confirm the diagnosis, molecular genetic testing should be performed to identify the homogenous or compound heterogenous mutated EPG5. Other useful tests for VICIS cases are ophthalmologic tests and ElectroencephalographyM, especially if seizures are present. Our patient underwent all these investigations.

The main causes of death among cases in the first or second year of life are heart failure and sepsis. Survival analysis shows that patients with VICIS have a median survival time period of 24 months (95% confidence interval, 0–39 months).^[3]^ Thus, therapeutic interventions for VICIS are mainly supportive and directed to relieve the symptoms and to improve the survival time.

Similarly, the most common causes of death among all reported patients with VICIS were recurrent infections and cardiomyopathy. Cardiac functions can deteriorate from the baseline into a heart failure in a short period of time, as shown for our patient. Therefore, patients with VICIS need to be monitored regularly for early detection of their VICIS-related complications, thus allowing timely intervention for a better outcome and prolonged survival.

In conclusion, VICIS should be considered in those children with multi-systemic involvement including central nervous system, cardiovascular system and developmental delay especially in Saudi Arabia due to high consanguineous marriages rates.

## Author contributions

KA: set the idea of the study and designed the study.

KA, NK, AB, MA, AA: reviewed literature, drafted the manuscript, critically analyzed the data.

All authors reviewed and approved the manuscript for final publication.
